# Swainson's thrushes vary singing behaviour across ambient noise gradients and rapidly adjust songs in response to experimental traffic noise

**DOI:** 10.1098/rsos.252553

**Published:** 2026-10-28

**Authors:** Brigitta Mathews, Riley Crawford, Annika Dank, Jasper McCutcheon, Cvetana Ilic, Christopher N. Templeton

**Affiliations:** ^1^ Department of Biology, Western Washington University, Bellingham, WA, USA; ^2^ Department of Cognitive Science, Pitzer College, Claremont, CA, USA

**Keywords:** bird song, urban ecology, anthropogenic noise, vocal communication

## Abstract

Anthropogenic noise pollution has a variety of negative consequences for animals, with observed effects ranging from physiological processes to community structures. Sound waves from anthropogenic noise can mask animal signals, creating challenges for animal communication. Songbirds have been observed to combat noise pollution by changing their song rate, duration, frequency or amplitude, with different species employing different strategies. We examined whether Swainson's thrushes (*Catharus usulatus*) adjust their songs under different traffic noise regimens using both population-level surveys and individual-level noise playback experiments. Swainson's thrushes' introductory note frequency varied with background traffic noise levels across the surveyed population, but other song parameters did not. When confronted with realistic levels of experimental traffic noise playback, individual thrushes rapidly changed the acoustic features of their songs, increasing the duration of their songs and adjusting the frequency parameters of the introductory notes before quickly returning towards baseline levels after the noise playback ended. Together, these results suggest that Swainson's thrushes adjust their singing to reduce acoustic masking in noisy areas and further indicate that some songbirds might continually assess ambient noise levels and only adjust their signals when necessary.

## 1. Introduction

Anthropogenic noise, which is noise produced by humans, has a wide variety of impacts on animals. While urban growth produces many types of pollution, anthropogenic noise is of particular importance owing to its ability to spread beyond areas immediately affected by urbanization to those without human development [1]. At the population and community levels, noise pollution can cause a reduction in biodiversity as usable habitats are altered and lost [1]. At the individual level, anthropogenic noise has negative implications for a variety of animal behaviours [2–6], including reproductive success [7,8], reduced predator defences [9], decreased cognitive performance [10,11] and changed foraging behaviours [12]. Unsurprisingly, some of the largest behavioural impacts of noise pollution are related to auditory communication [13].

Anthropogenic noise directly disrupts detection of auditory signals between conspecifics and heterospecifics through auditory masking [14,15]. For example, noise pollution disrupts the communication of anti-predator information in dwarf mongooses (*Helogale parvula*) through masking of surveillance calls [16]. Many species of marine mammals have also been observed to change their vocal signals in response to increasing levels of background noise. For example, right whales (*Eubalaena glacialis*) increase their call amplitudes in noisy ocean environments to avoid masking [17] and killer whales (*Orcinus orca*) increase the duration of their pod-specific social calls in response to increasing levels of boat traffic [18]. Similar patterns are also seen in many other species, including monkeys, bats and birds [19].

In avian species, anthropogenic noise has been found to impact acoustic features of songs in a variety of ways [3,20]. Alterations in a bird's songs can have detrimental consequences for mating behaviours, territory defence, energy usage and population density [15,21–23]. Consequently, understanding how anthropogenic noise impacts communication could help provide key insights into how noisy habitats impact avian fitness. Traffic noise is particularly detrimental to avian communication because of its high-energy, low-frequency noise profile, which can mask the vocalizations of many songbirds [24]. Species with lower natural vocalization frequencies are particularly vulnerable to the acoustic masking effect of traffic noise [3,25,26]. To compensate for acoustic masking from anthropogenic noise, birds have been shown to alter songs by changing four different components of their vocalizations: amplitude, duration, song rate and frequency [13].

Changes in amplitude are primarily attributed to the Lombard effect; first identified in 1911, this effect describes the phenomenon of ‘speaking up’ in noisy environments [19]. An increase in vocal amplitude as a response to the presence of background noise has been identified in a variety of species including humans, monkeys and whales [19]. Birds have been shown to alter the amplitude of their songs in a similar fashion. For example, a study of nightingales (*Luscinia megarhynchos*) across a natural ambient noise gradient revealed that birds in noisy habitats sang with higher amplitude than those in quieter habitats [27]. Similarly, great tits (*Parus major*) increased the amplitude of their alarm calls when faced with experimental noise in a controlled laboratory setting [28].

Birds have also been shown to adjust their song duration in the presence of noise. A study on great tits found that song lengths and inter-song intervals were shorter in city birds compared with their rural counterparts [29]. This could be a result of the non-forested urban habitat making it easier for songs to travel, causing birds to choose short songs to reduce the energetic cost of singing [29]. Alternatively, birds can avoid vocal masking by increasing signal redundancy, which can be achieved by increasing the song duration [30]. A study on yellow warblers (*Setophaga petechia*) inhabiting different islands in the Galapagos found that on an island with lower human activity and less anthropogenic noise, the warblers decreased their song duration in response to experimental noise playback. By contrast, on a noisier island, they increased song duration during noise exposure [31]. Similarly, a study on house finches (*Haemorhous mexicanus*) showed that the duration increased with lower-frequency notes but decreased with higher-frequency notes in response to noise playback [32]. Both studies support the idea that the relationship between duration and noise may depend on factors such as a bird's prior exposure to certain noise levels and the frequency of its vocalizations.

Similar to song duration and amplitude, the rate at which birds vocalize is altered in noisy environments [2,33]. Changes in song rate in response to increased anthropogenic noise have been observed in previous studies, but the changes are not consistent across species. A study comparing European robins (*Erithacus rubecula*) in noisy and quiet areas in Northern Ireland showed that birds inhabiting noisier areas sang fewer and less complex songs than those in quiet areas [34]. By contrast, a study investigating how the zebra finch responds to growing noise levels revealed that they increase their song rate up to an ambient noise threshold of 85 dB, at which point the rate begins to decrease or singing halts altogether [13]. These represent two different strategies that birds use to modulate song rate to promote communication during acoustic masking conditions. For some species, an increased song rate in noisier conditions may improve signalling by increasing the time spent singing, and therefore increasing signal redundancy [30], while a decreased song rate may be an attempt to avoid competition with low-frequency anthropogenic noise [35].

Finally, anthropogenic noise has been shown to affect song frequency. Common changes in song frequencies include spectral shifts and omission of low-frequency notes [29]. Spectral shift refers to either a frequency change in all notes within a vocalization or in only the lowest frequency notes [3,5,29]. Peak frequency, the frequency with the maximum amplitude in a signal, can vary if a bird sings a different song type or changes the spectral energy within the same song type without changing other features [26]. Bandwidth, the frequency range between the minimum and maximum frequencies, is a useful marker for comparing spectral shifts and low note omissions. The bandwidth of songs has been shown to decrease in noisy conditions, but this occurrence is not consistent across species and studies [36–38]. Another potential tactic for birds is to raise the minimum frequency of songs as ambient noise increases ([3,24]; but see [39] and [40]). When anthropogenic traffic noise decreased owing to COVID-19 lockdowns [41], birds sang songs with lower minimum frequencies and increased bandwidth [24]. This indicates that song frequency shifts can be plastic, potentially allowing birds to alter their signals to match the environmental conditions.

In this study, we investigated the impact of anthropogenic noise on the acoustic features of Swainson's thrush *(Catharus ustulatus*) songs. The Swainson's thrush has a distinct flute-like song that ascends in pitch as it progresses. The song begins with a low-frequency introductory note, followed by four to six syllables that are longer, louder and of higher frequency than the first note [42]. Breeding in coniferous forests, Swainson's thrushes are found in North America during spring and summer and migrate to Central and South America during the non-breeding season. During the breeding season, males sing regularly throughout the day to attract mates and to establish and defend territories. Swainson's thrushes have a repertoire of three to seven song types that are typically sung in a specific fixed order [42]. Because their breeding range overlaps with growing urban developments, Swainson's thrushes' exposure to anthropogenic noise is probably increasing in parallel with increases in urbanization across western Washington. Here, we examined whether anthropogenic noise impacts Swainson's thrush song features and singing behaviour to determine the potential short- and long-term impacts of noise pollution and strategies that this species uses to combat acoustic masking from noise pollution.

Following a protocol used by LaZerte *et al.* [33], we evaluated Swainson's thrush singing behaviour by combining population-level recordings of birds singing across a gradient of background noise levels and conducting experimental anthropogenic noise playbacks to identify rapid acoustic changes in individuals. To determine which strategies Swainson's thrushes are employing, if any, we analysed different song features, including song duration, song bandwidth, song rate and three measures of song frequency. We predicted that Swainson's thrushes would attempt to avoid the masking effects of low-frequency traffic noise by increasing minimum and peak frequencies and reducing the duration of songs as ambient noise levels increase, in both observational and experimental situations. We also predicted that owing to its low frequency, the introductory note of the Swainson's thrush song would show more pronounced effects, as it is likely to be most susceptible to masking.

## 2. Methods

### 2.1. Study sites

We studied Swainson's thrushes in locations within and nearby Bellingham, Washington, between 26 June and 8 August 2024. We recorded 44 individual vocalizing males in urban parks, green spaces and forests located in areas with varying levels of traffic noise (figure 1). Recordings were made between 8.00 and 13.00 during periods in which birds were naturally active and trials were not completed in inclement weather (rain, wind, etc.). Ambient noise levels at our study sites ranged from 30.5 to 73.3 dB (reference value for all sound pressure level (SPL) values in air is 20 µPa). Sites ranged from immediately adjacent to a busy interstate highway (I-5) to near-silent locations in pristine forests with minimal noise pollution, with specific sites chosen to capture a gradient of ambient anthropogenic noise across thrush breeding territories. All recordings were made during the breeding season, though the exact breeding stage for any given individual was unknown. Birds were not individually marked, but we ensured that the same individual was not recorded more than once by separating all focal individuals by at least 400 m. Previous studies examining territory size of marked Swainson's thrushes indicate very small breeding territories in this species [43], making 400 m more than sufficient to ensure that all birds we recorded were unique individuals.

**Figure 1.**
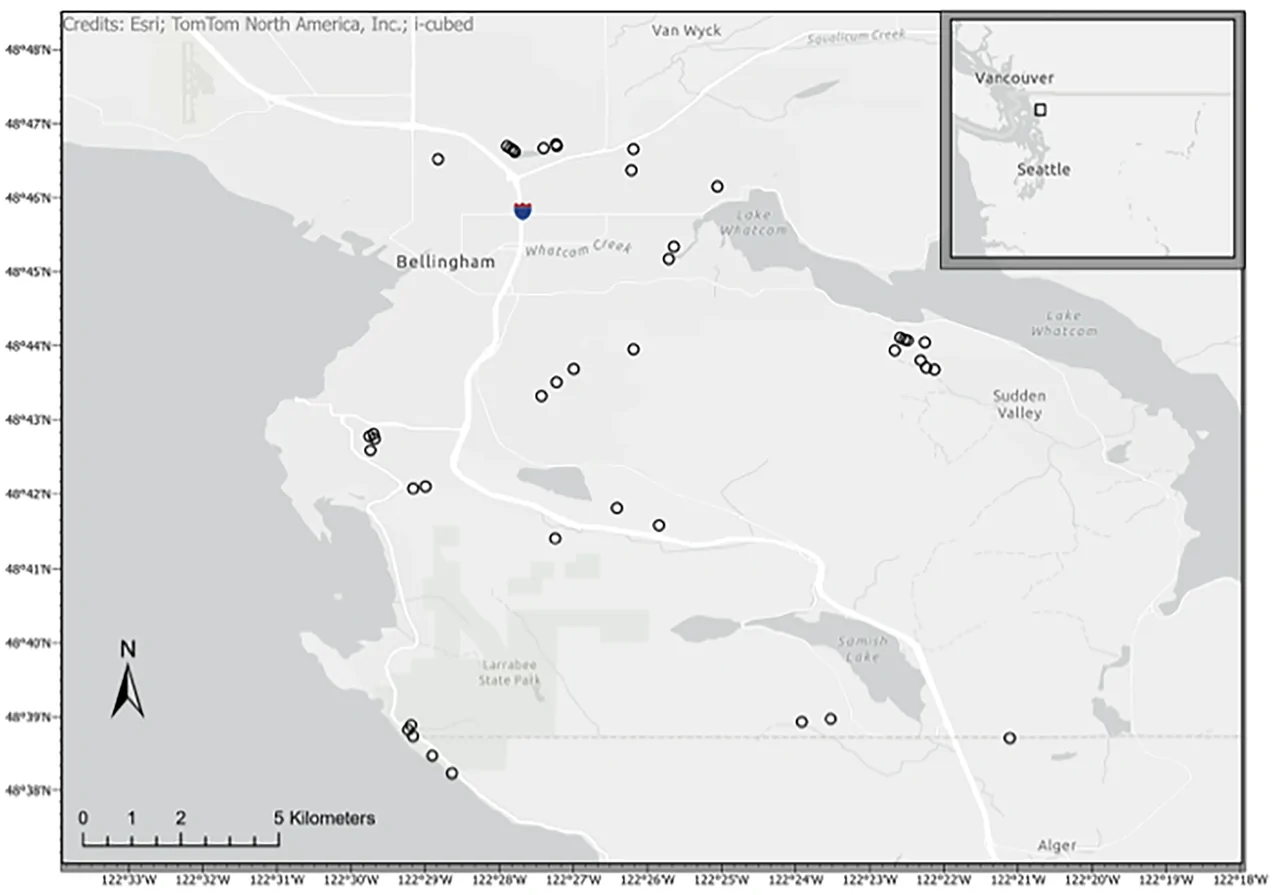
Study sites across western Washington, showing locations of focal birds (points), and major sources of traffic noise (white lines), including I-5 (freeway marker) and other highways.

### 2.2. Ambient noise measurements

We measured ambient noise levels at each site using an iPhone 13 with the NIOSH SPL app (threshold level = 80, exchange rate = 3 dB, time weighting = slow, frequency weighting = A) that was manually calibrated against a handheld SPL meter (Casella Cel-246). The NIOSH SPL meter app is advertised as being accurate to within 2 dB and previous research has shown similar, if not better, accuracy (e.g. [44,45]). To further confirm that our set-up was accurate enough for collecting dB readings in the field, we performed three validation tests (Templeton *et al.* 2026, unpublished data). First, we took 50 1-minute SPL readings across an amplitude gradient (30–80 dB) and compared this system with a dedicated SPL meter (Casella CEL-246, calibrated with CEL 120 Calibrator). We found that the dB readings from the iPhone NIOSH SPL app were very similar to those of the SPL meter, with less than 1 dB difference on average (0.99 ± 0.10 dB; average ± standard error (SE)) across all measured amplitude ranges. Next, to examine potential variation in performance across frequencies, we played single-frequency sine waves ranging from 200 Hz–2 kHz at 200 Hz steps under controlled lab conditions and used both devices to assess the SPL at amplitudes ranging from 40–80 dB, each at approximately 12 steps, again demonstrating that most frequencies had less than 2 dB difference between devices across all amplitudes, with slightly higher deviation (approx. 4 dB) between 1400 and 1600 Hz. The two devices responded similarly to changes in amplitude, with the comparison producing highly linear responses for each frequency across amplitudes (linear regression of Cel versus NIOSH SPL for each frequency tested: *R^2^* = 0.999–1.0), with NIOSH again reporting slightly lower dB for each frequency. Finally, we compared the dB readings between the two devices when assessing actual recordings of traffic noise presented at varying dB levels under controlled laboratory conditions. We recorded SPL on both devices in response to the same traffic noise files used in the playback study (details below) varying in the range 50–80 dB. Because actual traffic noise varies in amplitude across time, we used both devices to calculate a 30 s averaged dB level at each playback amplitude level, again demonstrating a highly linear response (*R^2^* = 0.998) with less than 2 dB difference between devices across all amplitudes. Together, these three validation studies help confirm that this NIOSH system was reliable and accurate enough for the purposes of this type of ecological study.

To assess the background amplitude at each location where Swainson's thrushes were recorded singing, we made two 60 s recordings 1.5 m above the ground and approximately 10 m from the focal individual, taking the average LAeq (dB) reading to account for natural variation in ambient noise. The first measurement was taken at the beginning of the survey period and the second measurement was taken towards the end of the survey. For birds that were exposed to experimental playback, the first measurement was taken in the pre-playback period and the second was taken in the post-playback period. A protocol mistake resulted in a single noise recording being taken for eight of the 44 birds, which was used in place of an average. These recordings occurred across a range of SPL values, so having only one measurement for these data points is unlikely to bias the results.

### 2.3. Population-level song survey

To assess how song parameters vary with background noise, we recorded birds across a traffic noise gradient. We recorded natural singing bouts for each male without the aid of playback to elicit songs. Recordings were taken of singing male Swainson's thrushes using Marantz PMD661 recording devices and Sennheiser ME66/K6 shotgun microphones approximately 10 m from the focal individual. Recorders were set to record uncompressed .wav files at a 44 kHz sampling rate. We recorded each focal individual for 5 minutes. We completed surveys for a total of 42 individual birds.

### 2.4. Individual responses to noise playback

To experimentally assess how individuals respond to varying traffic noise levels, we conducted a repeated measures playback experiment with individual Swainson's thrushes. If a bird was still vocalizing at the end of the 5-minute survey period (‘pre-playback’), we then exposed it to a 5-minute recording of traffic noise (‘noise playback’). Experimental traffic noise was recorded from a moderately busy highway and provided realistic levels of highway noise [10,28]. These recordings all included natural variations in traffic noise of cars and were normalized so that they all had the same peak amplitude [28]. We calibrated a FoxPro Crossfire speaker using a CEL 246 SPL meter so that the playback would add approximately 65 dB of anthropogenic noise during playback when the speaker was placed *ca* 10 m from the focal bird at 1–2 m height. This playback amplitude was chosen to provide a realistic level of traffic noise and matches amplitudes used in previous studies [33]. We randomly selected one of five different exemplar noise recordings to play at each site using a random number generator (each exemplar was used on average 3 ± 0.5 times; mean ± SE). After the noise playback finished, we continued recording for 5 minutes (‘post-playback’). We conducted experimental trials with 15 individuals (*N* = 15 for song rate data and *N* = 14 for song structure data, as one bird did not sing during the playback period).

### 2.5. Acoustic analysis

Analysis of the high-quality audio recordings was performed using Raven Pro v.1.5 software (Cornell University, Ithaca, NY, USA) to measure our chosen variables for each individual Swainson's thrush. The Raven selection tool was used to visually select up to 10 full songs from each survey recording and up to 10 from each of the playback periods for experimental trials (pre-, during noise and post-playback). Full songs included all phrases of an individual's song, including the introductory note (figure 2). Some recordings did not have 10 high-quality songs available for selection owing to overlap of other vocalizations, loud background noises or poor audio quality (minimum = 3, average = 8 songs used per individual). Because of their relatively low frequency, we also separately identified and selected the introductory notes of each song. Introductory notes are typically the lowest notes in a Swainson's thrush song [42], with a high degree of overlap with traffic noise, making them potentially susceptible to masking. Selection bias was reduced by following a uniform protocol [46], with measurements taken from spectrograms set to the following parameters: window type: Hann, window size: 1024 Hz, 3 dB filter bandwidth: 67.4 Hz, brightness: 75 and contrast: 65 [46]. We used Raven Pro to calculate acoustic parameters that were biologically meaningful and had been previously documented in other studies examining noise pollution and bird song (e.g. [33]). To measure the minimum and maximum frequencies, we used frequencies of 5% (Hz) and 95% (Hz). We used duration of 90% (s) to measure the duration of each song and introductory note and calculated bandwidth as the difference between frequencies of 95% and 5%. These measurements are considered to be robust signal measurements and are therefore less sensitive to small variations in the selection measurements [39,41,46]. Finally, we used peak frequency (Hz) to measure the frequency at which the sound had the most energy. Song vocalizations selected from the pre-playback segment of the experimental trials were also used as survey data points for applicable individuals, as the birds vocalizing in this phase of the experiment had not yet been exposed to experimental manipulations. To calculate song rate, every individual song was counted from the pre-, during noise and post-playback periods of each experimental noise trial and we calculated the average songs per minute for each trial period.

**Figure 2.**
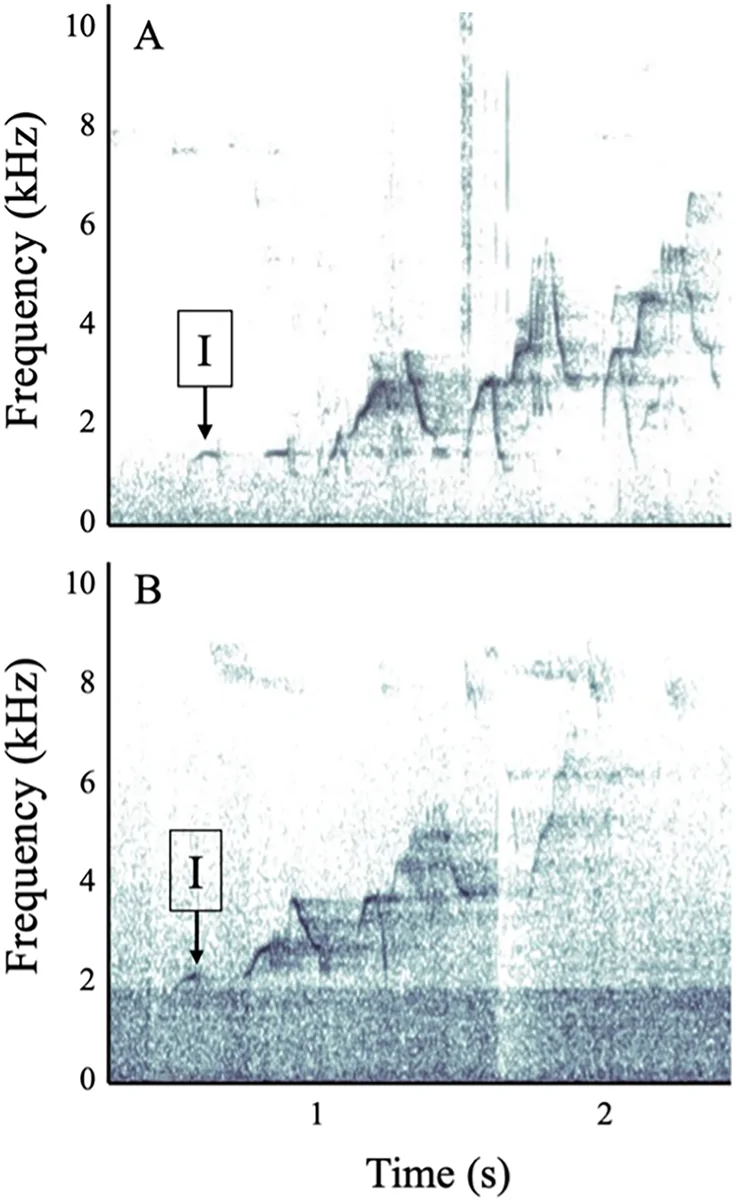
Swainson's thrushes sing an ascending and melodious song. Songs were recorded in both (A) relatively quiet locations and (B) locations with higher levels of background traffic noise. Song features were measured both for the entire song and for the introductory syllable, designated with an I.

### 2.6. Statistical analyses

To assess the impact of anthropogenic noise on Swainson's thrush song frequency, duration and rate, we created and ran linear mixed effect models (LMMs) using the lme4 R package in R v.2024.12.0+467 [47,48]. We fit LMMs using restricted maximum likelihood (REML), as this approach performs better than maximum likelihood with small sample sizes [49]. We used a combination of Shapiro–Wilk W tests, Levene's tests and visual inspections of diagnostic plots to assess whether the model assumptions were met [50], including normality of the random effect, normality of residuals, residual homoscedasticity and the independency of the residuals. The diagnostic plots we created to decide whether our models met the assumptions were: a residuals versus fitted values plot, a QQ plot for normality of residuals, a QQ plot for normality of random effects and an autocorrelation function plot.

We extracted *p*-values using the lmerTest package [51] to assist in determining the statistical significance of the models. The critical value for all comparisons using two-tailed tests was 0.05. The lmerTest package uses *t*-tests with Satterthwaite's approximation to provide *p*-values for LMMs [51]. We chose this method because of its applicability to REML models and reliability at assessing significance in LMMs [49]. We applied the Holm–Bonferroni correction [52] to the *p*-values extracted from our models to reduce the overall false-positive rate from multiple comparisons using the p.adjust() function from the stats package [48]. In addition, we calculated the *R^2^* values for our models using the performance package [53]. This allowed us to draw conclusions about the degree of biological significance of our results. This package calculates the marginal *R^2^* values with the approach from Nakagawa *et al.* [54], which we used to assess the contributions of each fixed effect.

#### 2.6.1. Population-level song survey

We ran an LMM for each combination of vocalization type (full song or intro note) and response variable (minimum frequency, maximum frequency, bandwidth, peak frequency and duration), which resulted in 10 unique models. The predictor variable was always ambient noise level (dB). We recorded songs from 42 unique individuals; for the models with the full song vocalization type, we included 328 songs, while the models with the intro note type included 357 notes. To remove intra-individual variation from our data, we set the individual ID (a unique identifier for each bird) as a random effect. Of the 10 total models, all but two met the assumptions of LMMs. The two problematic models failed to meet the residual homoscedasticity assumption. Because the heteroscedasticity in those models was not dramatic and linear mixed models are generally robust to violations of this assumption [50,55], we felt it was acceptable to move forward with those models. Adding the time of day in which the recordings were made as a covariate did not improve the overall model fit; so, this variable was not included in the final models.

#### 2.6.2. Individual responses to noise playback

We also used LMMs to analyse the experimental traffic noise playback data. There were 11 models in total, each representing a unique combination of a vocalization type (full song or intro note) and a response variable. These variables were minimum frequency, maximum frequency, bandwidth, peak frequency, duration and song rate, with the latter only used for the full song vocalization type. The predictor variable for all these models was the playback phase (pre-, during noise or post-playback). We recorded songs from 15 unique individuals in each phase of the study; models with the full song vocalization type had a sample size of 119 pre-, 62 during noise and 90 post-playback songs, while the intro note type had 126 pre-, 64 during noise and 90 post-playback songs. For this suite of models, we also removed intra-individual variation from our data by using individual ID as a random effect. We concluded that all 11 models met the assumptions of LMMs following a visual inspection of the diagnostic plots of each.

To quantify how Swainson's thrushes varied their song characteristics during the different portions of traffic noise playback, we calculated estimated marginal means (EMMs). These values represented how much the vocalizations of Swainson's thrushes changed in response to playback. Here, EMMs indicate the predicted average values of the response variables after accounting for individual variation and were calculated using the emmeans R package [56]. Adding the time of day at which the recordings were made and playback exemplar number as covariates did not improve the overall model fit; so, these variables were not included in the final models.

## 3. Results

### 3.1. Population-level song survey

Swainson's thrushes' song features varied along a noise gradient, with most effects occurring to the low-frequency introductory notes (table 1). Survey data showed that ambient noise levels had little overall effect on the minimum frequency, maximum frequency, frequency bandwidth, peak frequency or song duration (*p* > 0.05, *R^2^* < 0.03; figure 3). By contrast, ambient traffic noise levels had a strong effect on the low-frequency introductory note of Swainson's thrushes' songs (table 1 and figure 4). Analysis of intro notes showed that the minimum frequency (10.81 ± 2.91 Hz dB^–1^; *p* = 0.005; figure 4A) increased comparably with the maximum frequency (12.53 ± 3.61 Hz per 1 dB increase; *p* = 0.009; figure 4B). Similarly, the peak frequency of the intro notes was higher as the noise increased (*p* = 0.005; figure 4D). The bandwidth (*p* = 1.000; figure 4C) and duration (*p* = 1.000; figure 4E) of intro notes did not significantly vary with noise levels. The variation seen in the minimum, maximum and peak frequency was moderately explained by ambient noise (marginal *R^2^* = 0.123, 0.113 and 0.115, respectively), which only poorly explained variation in bandwidth (*R^2^* = 0.007) and duration (*R^2^* = 0.003).

**Table 1.** LMM results for the impact of ambient noise on Swainson's thrushes' full song and intro note features. Degrees of freedom (d.f.) and *t*-values were derived using the Satterthwaite's approximation and adjusted *p*-values used the Holm–Bonferroni correction. Bold text indicates significant effects (determined using adjusted *p*-value).

response variable	parameter	estimate	SE	d.f.	*t*-value	*p*-value
full song minimum frequency (Hz)	intercept	2169.15	151.66	45	14.30	<0.001
	ambient noise (dB)	–4.65	3.20	45	–1.46	1.000
full song maximum frequency (Hz)	intercept	4468.42	419.54	45	10.65	<0.001
	ambient noise (dB)	–3.71	8.83	45	–0.42	1.000
full song bandwidth (Hz)	intercept	2277.57	382.28	45	5.96	<0.001
	ambient noise (dB)	1.44	8.05	46	0.18	1.000
full song peak frequency (Hz)	intercept	2954.17	228.96	48	12.90	<0.001
	ambient noise (dB)	–1.45	4.84	49	–0.30	1.000
full song duration (s)	intercept	1.03	0.15	44	6.80	<0.001
	ambient noise (dB)	0.00	0.00	45	0.68	1.000
intro note minimum frequency (Hz)	intercept	1134.88	138.46	49	8.20	<0.001
	**ambient noise (dB)**	**10.81**	**2.91**	**50**	**3.71**	**0.005**
intro note maximum frequency (Hz)	intercept	1477.10	171.69	47	8.60	<0.001
	**ambient noise (dB)**	**12.53**	**3.61**	**48**	**3.47**	**0.009**
intro note bandwidth (Hz)	intercept	321.04	114.45	47	2.81	0.007
	ambient noise (dB)	2.14	2.41	48	0.89	1.000
intro note peak frequency (Hz)	intercept	1366.64	157.02	46	8.70	<0.001
	**ambient noise (dB)**	**12.31**	**3.31**	**47**	**3.72**	**0.005**
intro note duration (s)	intercept	0.09	0.02	47	5.19	<0.001
	ambient noise (dB)	0.00	0.00	48	0.68	1.000

**Figure 3.**
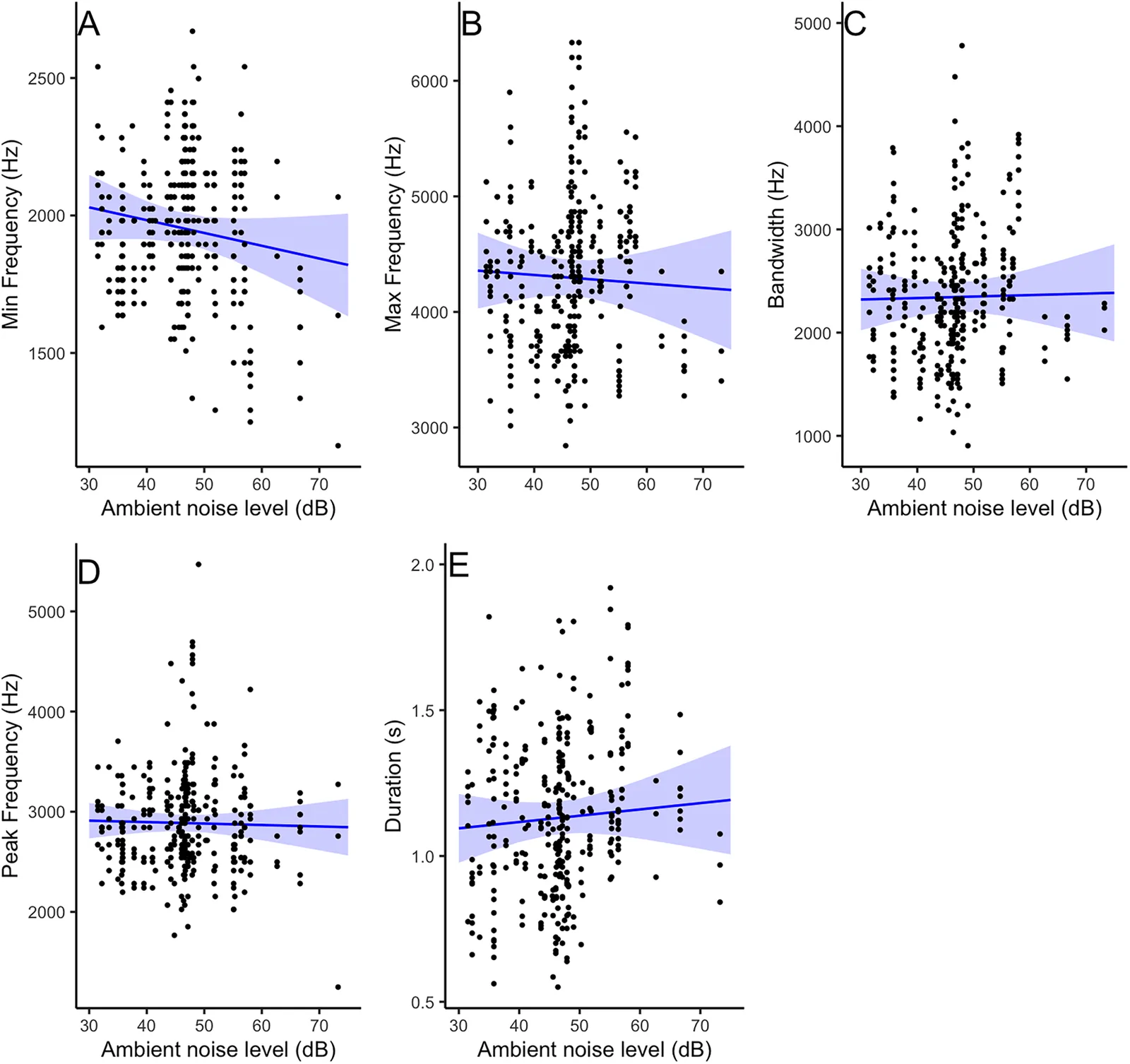
There were no significant patterns between (A) minimum frequency, (B) maximum frequency, (C) bandwidth, (D) peak frequency and (E) duration of Swainson's thrushes' songs and the ambient noise levels. Points represent raw data while the blue line and shaded areas indicate LMM 95% confidence intervals.

**Figure 4.**
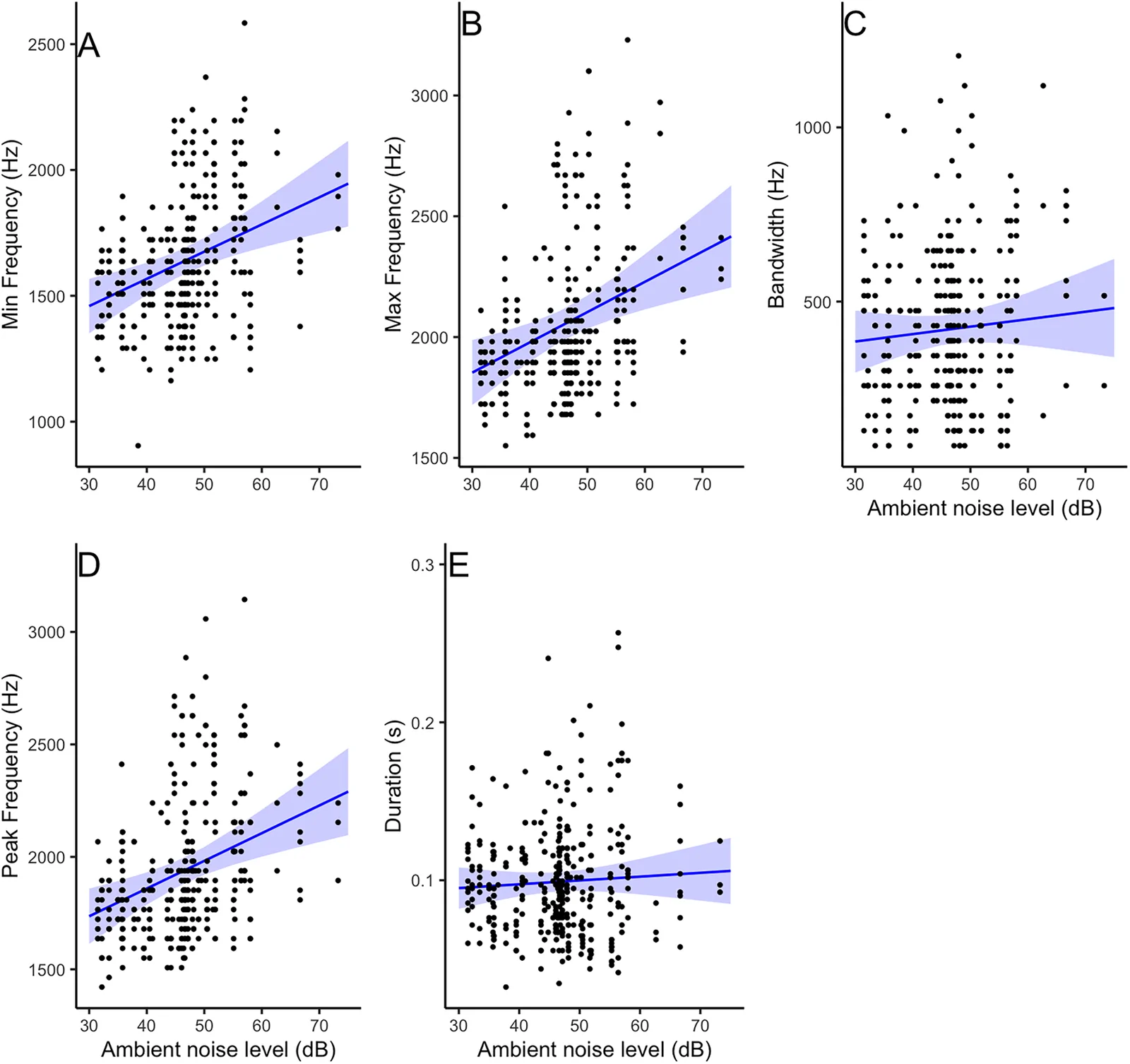
In areas with greater ambient noise levels, Swainson's thrushes' songs had intro notes with higher (A) minimum, (B) maximum and (D) peak frequencies, while their (C) bandwidth and (E) duration showed no patterns. Points represent raw data with the blue line and shaded areas indicate the estimates and 95% confidence intervals from the respective LMMs.

### 3.2. Individual responses to noise playback

Individual Swainson's thrushes rapidly changed the duration of their songs when confronted with the experimental traffic noise playback (table 2). Specifically, Swainson's thrushes increased the length of their songs in response to noise playback, with song length returning to pre-playback levels after the noise playback ended (noise, *p* < 0.001; post, *p* = 1.000; figure 5E). Minimum frequency, maximum frequency, bandwidth, peak frequency and song rate did not significantly change between the different playback phases (figure 5A–DF). The playback of traffic noise poorly explained the remaining variation for minimum frequency, maximum frequency, bandwidth, peak frequency, duration and song rate (marginal *R^2^* = 0.001, 0.000, 0.000, 0.005, 0.061 and 0.050, respectively). While there was some variation in responses across different individuals, most responded to noise playback in a similar fashion ( figure 7, appendix A).

**Table 2.** EMMs results from a LMM for the impact of experimental noise playback on Swainson's thrushes' full song and intro note features. Degrees of freedom (d.f.) and *t*-values were derived using the Satterthwaite's approximation and adjusted *p*-values used the Holm–Bonferroni correction; *t*-tests compared the during noise and post periods to the reference level (pre). Bold text indicates significant effects (determined using adjusted *p*-value).

response variable	parameter	estimate	SE	d.f.	*t*-value	*p*-value
full song minimum frequency (Hz)	pre (intercept)	2000.97	34.75	19	57.58	<0.001
	noise	1987.61	40.60	267	–0.39	1.000
	post	1986.32	37.23	268	–0.48	1.000
full song maximum frequency (Hz)	pre (intercept)	4268.33	125.78	14	33.93	<0.001
	noise	4272.35	135.41	261	0.05	1.000
	post	4261.93	129.85	263	–0.09	1.000
full song bandwidth (Hz)	pre (intercept)	2266.99	116.37	15	19.48	<0.001
	noise	2285.53	126.96	262	0.23	1.000
	post	2277.92	120.85	264	0.15	1.000
full song peak frequency (Hz)	pre (intercept)	2877.12	60.86	27	47.28	<0.001
	noise	2973.27	79.32	261	1.14	1.000
	post	2925.59	68.51	260	0.65	1.000
full song duration (s)	pre (intercept)	1.10	0.05	16	22.07	<0.001
	**noise**	**1.28**	**0.06**	**265**	**4.25**	**<0.001**
	post	1.10	0.05	267	–0.09	1.000
full song rate (songs/minute)	pre (intercept)	4.03	0.71	40	5.64	<0.001
	noise	2.61	0.71	28	–1.52	1.000
	post	3.85	0.71	28	–0.19	1.000
intro note minimum frequency (Hz)	pre (intercept)	1625.93	37.82	16	42.99	<0.001
	**noise**	**1981.51**	**42.37**	**271**	**11.89**	**<0.001**
	**post**	**1729.29**	**39.94**	**276**	**3.74**	**0.004**
intro note maximum frequency (Hz)	pre (intercept)	1947.11	45.26	17	43.02	<0.001
	**noise**	**2382.32**	**51.71**	**272**	**11.08**	**<0.001**
	post	2046.93	48.26	277	2.75	0.104
intro note bandwidth (Hz)	pre (intercept)	321.22	22.95	24	14.00	<0.001
	noise	398.48	28.85	277	2.76	0.104
	post	316.36	25.70	273	–0.19	1.000
intro note peak frequency (Hz)	pre (intercept)	1854.43	46.45	17	39.92	<0.001
	**noise**	**2273.16**	**52.83**	**272**	**10.62**	**<0.001**
	post	1944.04	49.42	276	2.46	0.217
intro note duration (s)	pre (intercept)	0.09	0.00	23	20.73	<0.001
	noise	0.10	0.01	276	1.96	0.714
	post	0.09	0.00	275	–0.07	1.000

**Figure 5.**
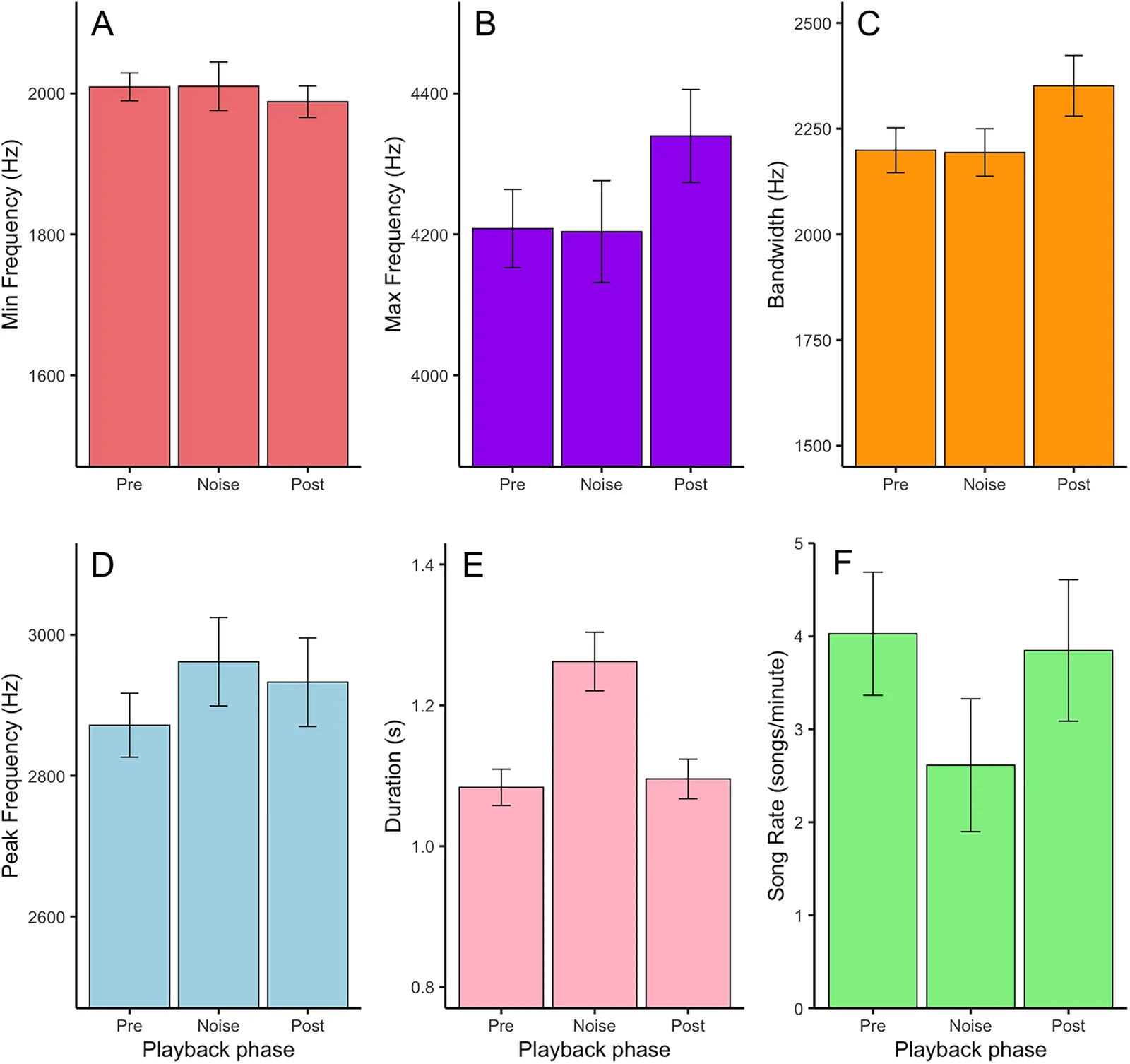
Swainson's thrushes rapidly changed their song duration, but not other acoustic features of their songs, upon exposure to the experimental traffic noise playback. Mean ± SE is shown for the (A) minimum frequency, (B) maximum frequency, (C) bandwidth, (D) peak frequency, (E) duration and (F) song rate of Swainson's thrushes' full songs during the three playback phases (pre, noise and post).

We found that the lower-frequency introductory notes were heavily affected by noise playback ( table 2). Swainson's thrushes significantly increased the minimum (*p* < 0.001; post, *p* = 0.004; figure 6A), maximum (*p* < 0.001; figure 6B) and peak frequency (*p* < 0.001; figure 6D) of their intro notes during noise playback and afterwards. The increase of these vocalization characteristics was relatively stronger during noise playback compared to after playback. Bandwidth and duration showed non-significant trends, with bandwidth (*p* = 0.104; figure 6C) and duration (*p* = 0.714; figure 6E), though the latter apparent pattern in the mean data did not approach statistical significance. The traffic noise playback explained a meaningful proportion of the variation in the minimum, maximum and peak frequency (marginal *R^2^* = 0.280, 0.268 and 0.250, respectively) of intro notes while poorly explaining that of bandwidth and duration (*R^2^* = 0.032 and 0.016, respectively). While there was some variation in introductory syllables across different individuals, most birds responded to noise playback in a similar fashion (figure 8, appendix A).

**Figure 6.**
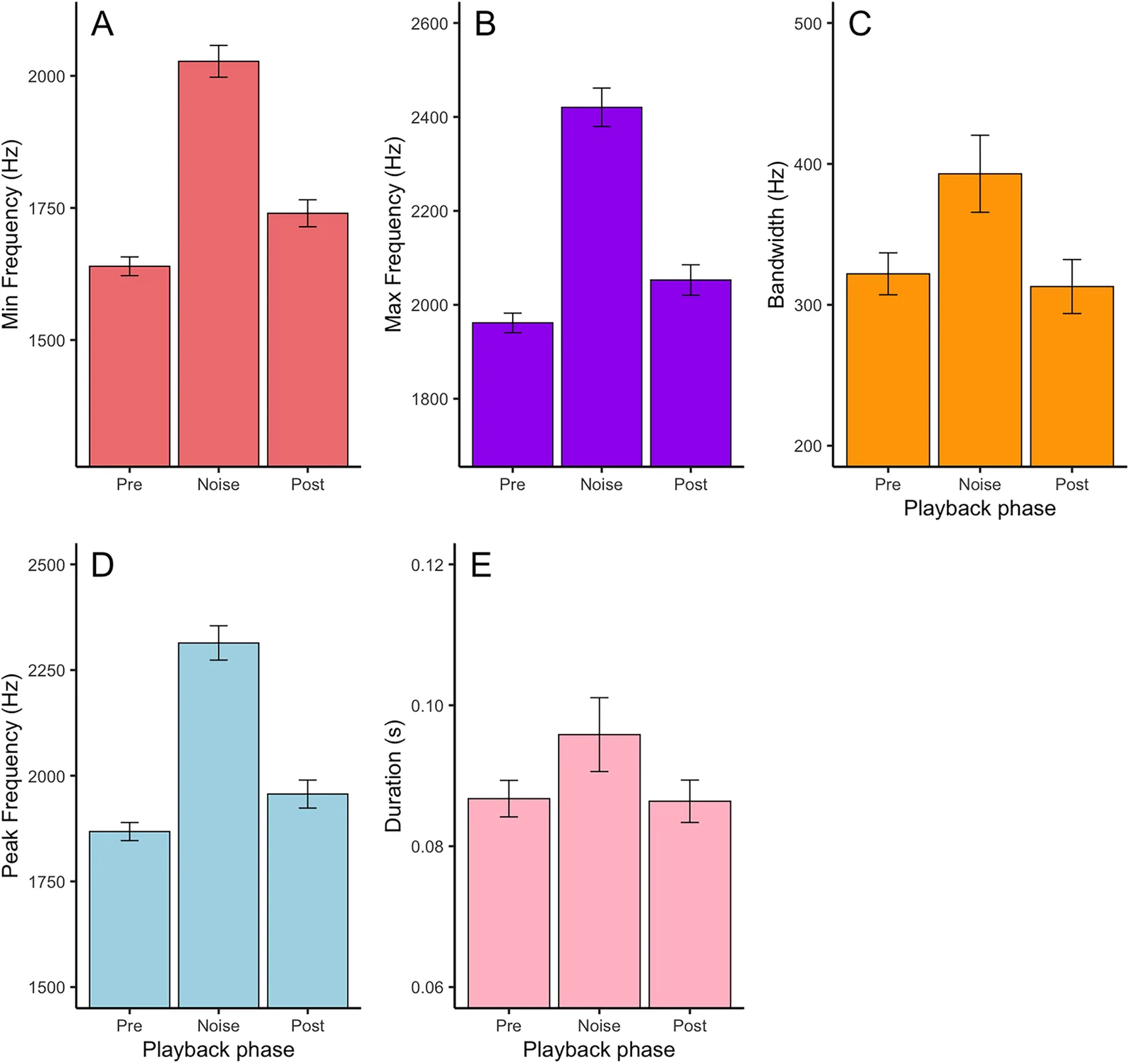
Swainson's thrushes rapidly changed the minimum frequency, maximum frequency, bandwidth and peak frequency of the intro notes of their songs upon exposure to the experimental traffic noise playback. Mean ± SE is shown for the (A) minimum frequency, (B) maximum frequency, (C) bandwidth, (D) peak frequency and (E) duration of Swainson's thrushes' intro notes during the three playback phases (pre, noise and post).

## 4. Discussion

We investigated whether Swainson's thrushes vary their songs and singing behaviour in response to anthropogenic noise by analysing frequency, duration, bandwidth and song rate with respect to population-level variation across an ambient noise gradient and through short-term experimental exposure of individuals to traffic noise playback. Our results showed significant relationships between acoustic features of Swainson's thrushes' songs and anthropogenic noise levels, in both the population-level survey and individual-level experimental data. These results indicate that Swainson's thrushes respond to anthropogenic noise exposure by varying their singing behaviour and song selection, with the most prominent changes being frequency shifts in the low-frequency introductory notes and a reduction of the duration of songs when exposed to anthropogenic noise. Together, these results suggest that Swainson's thrushes employ a variety of mechanisms to avoid auditory masking from low-frequency traffic noise.

Results from both the population- and individual-level components of the study showed an increase in minimum, maximum and peak frequencies in the introductory notes. Introductory notes are the lowest frequency elements in the ascending progression of Swainson's thrush songs [42], meaning that they have a large degree of spectral overlap with traffic noise and are particularly vulnerable to masking. This result is consistent with other studies that showed an increase in frequency of the introductory or lowest notes in low-frequency songs [57]. These results highlight the plasticity of the introductory note, which may be a functionally significant part of the Swainson's thrush song. Previous studies have hypothesized that intro notes are used for detectability or individual recognition in other species [58]. Our results show that Swainson's thrushes have the ability to alter these notes in response to environmental noise conditions, indicating plasticity in these learned and biologically predisposed song features.

While the frequency changes of the introductory notes under noise followed patterns described in other studies, the full song frequency parameters did not significantly shift with increased noise levels as has been observed in a number of similar studies (e.g. [15,24]). One potential explanation for this finding could be the frequency range of the main phrase of the Swainson's thrush song, as it contains higher-frequency components [42] that would not be as susceptible to masking as the low-frequency intro note. Another possible explanation could be that Swainson's thrush song components encode different information and therefore use different mechanisms to respond to increased noise levels. For example, a study on savannah sparrow (*Passerculus sandwichensis)* songs found that introductory notes, which announce the beginning of the song, and buzz components, which act as species-specific identifiers, were altered in the presence of industrial noise, but other syllables were not affected [59].

Amplitude has been shown to increase in response to noise in similar studies [27], though this was not directly analysed here. Further research into the Lombard effect [19] in Swainson's thrush songs could provide more insight into the frequency shifts observed in introductory notes. A study on blackbirds (*Turdus merula*) found that peak frequency and amplitude increased together in louder city environments, and this increased amplitude of the higher-frequency song elements had a greater effect on the reduction of masking [26]. In addition, there may be certain physical limitations that prevent increased amplitude of low-frequency vocalizations, with song elements including peak frequencies around 2 kHz and below, such as the Swainson's thrush introductory notes, being particularly difficult to produce at high amplitudes [26]. It is possible that increased road noise levels caused Swainson's thrushes to sing louder in an attempt to improve signal transmission, and in turn, increase the frequency of their introductory notes.

At the population level, Swainson's thrushes showed no significant change in song duration. However, at the individual level, they increased the full song duration in response to noise playback. Previous studies have found conflicting results regarding changes to song duration, which probably means that duration changes are species-dependent and possibly influenced by other factors, such as frequency of vocalization, habitat and type of noise pollution [25,32]. Similar studies that found increases in song duration propose that increasing song duration is an immediate strategy to compete with a novel noise [30,33]. Similarly, the immediate increase in Swainson's thrush song duration shown by individuals confronted with experimental traffic noise could be an attempt to communicate over the noise, indicating short-term behavioural plasticity which would be unsustainable for longer periods of noise disturbance. Further work examining why Swainson's thrushes did not demonstrate such plasticity at the population level may elucidate the long-term effects of anthropogenic noise on songbirds.

Results for song rate were not significant in this study, though we did observe a general trend of decreased singing during experimental playback compared to the pre- and post-playback periods. It is possible that Swainson's thrushes are assessing noise-level variations and waiting for a quieter opportunity to sing. A study investigating the relationship between daytime noise levels and European robins' (*E. rubecula)* singing behaviour found that robins sang nocturnally in areas with higher daytime noise levels, probably to take advantage of quieter conditions or avoid competition from anthropogenic noise [35]. Further research should investigate whether Swainson's thrushes shift from songs to calls during noise exposure, a pattern that has been observed in other species [33].

While Swainson's thrushes are still a relatively common species in northern forests, their population has declined in many places, probably in part owing to the loss of important habitats such as mature forests and riparian zones. One study found that out of 244 Swainson's thrushes nesting in British Columbia, Canada, 84% nested in undisturbed forest area [60]. We observed a similar trend during our data collection, generally finding more vocalizing males in relatively quieter areas than in very loud roadside habitats (pers. obs.). Even with their apparent ability to immediately adjust acoustic features, Swainson's thrushes may still seek out pristine habitats and avoid noisier locations, implying that noise impacts their assessment of habitat quality [61]. The impacts of noise pollution have many detrimental effects on avian communication. Recent literature points to potential costs of changing song features in response to noise, including impaired territorial interactions between males, increased risk of predation or parasitism and reduced clutch sizes in noisy nesting areas [24,25,62]. Investigating the survival and reproduction rates of Swainson's thrushes in environments with varying noise levels would give insight into longer term impacts of anthropogenic noise.

Our findings show that Swainson's thrushes' songs are influenced by anthropogenic noise levels, with individuals appearing to possess the ability to adjust their singing behaviour in response to short-term bursts of traffic noise. Population-level changes in the minimum, maximum and peak frequencies of intro notes indicate that Swainson's thrushes are among the species that have the vocal plasticity to assess variation in noise levels and adjust their vocalizations. In addition, the lack of change in the full song at the population level suggests that Swainson's thrushes may be among the species that alter only certain parts of their song in response to anthropogenic noise. While we observed significant effects of noise pollution on a variety of song features at the individual and population level, there was variation in the degree to which noise explained song parameters. The estimated *R^2^* values indicated that noise alone explained less than 50% of the overall variation, suggesting that a substantial amount of variation in song features was not accounted for by noise alone. Future studies should examine other potential ecological drivers of different song features to better understand this variation. Together, our results add to the growing body of research demonstrating that anthropogenic noise impacts avian communication and highlight the diverse strategies that animals use to overcome communication challenges.

## Figures and Tables

**Figure 7.**
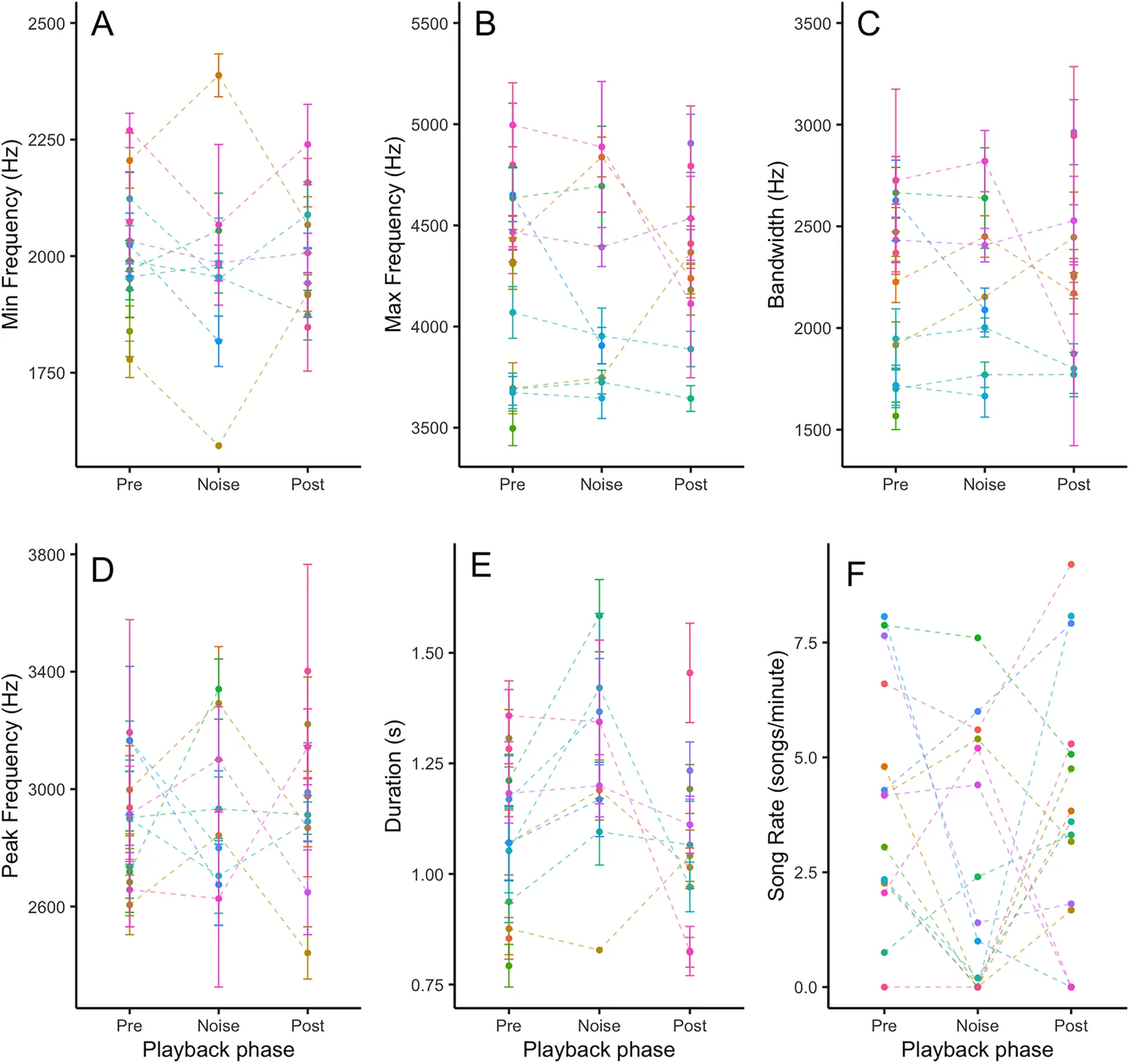
Swainson's thrushes significantly changed the duration of their songs upon exposure to experimental traffic noise playback. The mean ± SE for each individual bird is shown for (A) minimum frequency, (B) maximum frequency (C) bandwidth, (D) peak frequency and (E) duration, along with the (F) total song rate (songs/minute) of Swainson's thrushes' full songs during the three playback phases (pre, noise and post). Each colour represents an individual Swainson's thrush with lines connecting their response in each playback phase. Some individuals sang only in one or two of the three phases. There are no connecting lines for the individuals who vocalized in the pre- and post-playback periods but not the during noise period.

**Figure 8.**
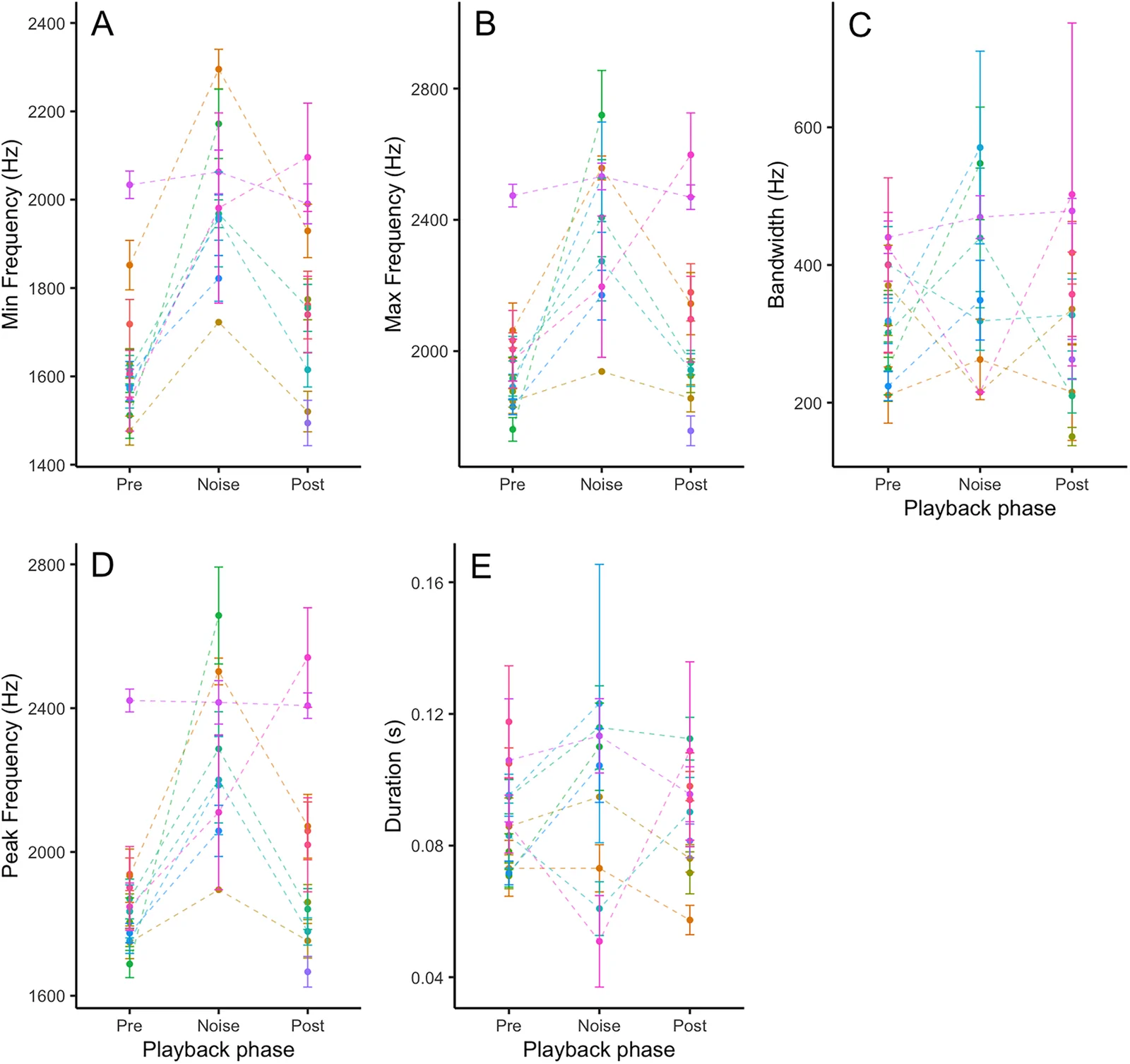
Swainson's thrushes significantly changed the minimum, maximum and peak frequencies of the introductory notes of their songs upon exposure to experimental traffic noise playback. The mean ± SE for each individual bird is shown for the (A) minimum frequency, (B) maximum frequency (C) bandwidth, (D) peak frequency and (E) duration of Swainson's thrushes' intro notes during the three playback phases (pre, noise and post). Each unique colour represents an individual Swainson's thrush with lines connecting their vocal response in each playback phase. Some individuals vocalized only in one or two of the phases. To avoid misinterpretation, there are no connecting lines for the individuals who only vocalized during pre- and post-playback.

## Data Availability

Data and relevant code for this research work are stored in GitHub: https://github.com/christempleton/Mathews_SWTH_song_noise/releases/tag/v1 and have been archived within the Zenodo repository [63].
